# State of the art: leadless ventricular pacing: A national expert consensus of the Austrian Society of Cardiology

**DOI:** 10.1007/s10840-019-00680-2

**Published:** 2019-12-20

**Authors:** C. Steinwender, P. Lercher, C. Schukro, H. Blessberger, G. Prenner, M. Andreas, J. Kraus, M. Ammer, M. Stühlinger

**Affiliations:** 1 Department of Cardiology and Internal Intensive Medicine, Kepler University Hospital, Johannes Kepler University Linz, Medical Faculty, Linz, Austria; 2 Department of Cardiology, Medical University Graz, Graz, Austria; 3 Department of Internal Medicine II, Division of Cardiology, Medical University of Vienna, Vienna, Austria; 4 Department of Surgery, Division of Cardiac Surgery, Medical University of Vienna, Vienna, Austria; 5 Universitätsklinik für Innere Medizin II, Cardiology, Paracelsus Medical University Salzburg, Salzburg, Austria; 6 Cardiology Department, Klinikum Wels-Grieskirchen, Wels, Austria; 7 University Clinic of Internal Medicine III, Cardiology and Angiology, Medical University of Innsbruck, Innsbruck, Austria

**Keywords:** Leadless pacing, Pacemaker, Cardiac implantable electronic devices, CIED, Bradycardia, Infection

## Abstract

**Background:**

Cardiac pacing has been shown to improve quality of life and prognosis of patients with bradycardia for almost 60 years. The latest innovation in pacemaker therapy was miniaturization of generators to allow leadless pacing directly in the right ventricle. There is a long history and extensive experience of leadless ventricular pacing in Austria. However, no recommendations of national or international societies for indications and implantation of leadless opposed to transvenous pacing systems have been published so far.

**Results:**

A national expert panel of skilled implanters gives an overview on the two utilized leadless cardiac pacing systems and highlights clinical advantages as well as current knowledge of performance and complication rates of leadless pacing. Furthermore, a national consensus for Austria is presented, based on recent studies and current know-how, specifically including indications for leadless pacing, management of infection, suggestions for qualification, and training of the operators and technical standards.

**Conclusions:**

Leadless pacing systems can be implanted successfully with a low complication rate, if suggestions for indications and technical requirements are followed.

**Condensed abstract:**

An overview of the two utilized leadless cardiac pacing systems is given, specifically highlighting clinical advantages as well as current knowledge of performance and complication rates. Furthermore, a national consensus for Austria is presented, specifically including indications for leadless pacing, management of infection, and suggestions for qualification and technical standards.

## Background: current clinical evidence for leadless pacing

Implantation of a cardiac pacemaker (PM) is a first-line therapy in symptomatic patients with bradyarrhythmias, as this intervention improves quality of life in sick sinus syndrome as well as in atrial fibrillation (AF) with slow atrioventricular conduction and additionally reduces mortality in patients with atrioventricular block [1, 2]. A remarkable advance in technology can be acknowledged since the first PM implantation in 1958 by Senning and Elmquist at the Karolinska Hospital in Stockholm [3]. Battery longevity of more than 10 years became a reality, the size of devices was reduced significantly, and it is meanwhile possible to program more than 150 parameters according to specific patient requirements. Currently, approximately 1 million transvenous PMs are implanted worldwide every year [4]. Despite these extraordinary improvements, PM therapy still has several limitations: Complications occur in approximately 9–12% of all patients, including pocket hematomas or pocket infections, lead fractures, or lead endocarditis, as well as pulse generator problems [5, 6]. Furthermore, PM electrodes may also cause venous obstruction and severe tricuspid regurgitation during long-term follow-up [7, 8].

Indeed, the transvenous lead is the weakest link of a PM system and is therefore considered the “Achilles heel” of this therapy. Firstly, venous obstruction after device implantation is not negligible, as total occlusions are detected in roughly 9% of patients [9, 10]. Although most of these cases are clinically silent, potentially necessary future lead revisions become a challenge and often require a change towards the contralateral side or an alternative venous or epicardial access. Secondly, transvenous lead endocarditis is a rare but serious complication and has been reported in 0.5–1.0% of patients within the first 12 months after implantation [5, 11, 12]. If a complete explantation of the pacing system is necessary in this respect, mortality rises significantly up to 20–30% of affected patients [13–16]. Thirdly, in the long-term, PM leads are prone to failure such as lead fracture or insulation break. The incidence of chronic lead failure is about 1–4% and the median time to failure 5 to 7 years [6, 17–20]. Lastly, lead dislodgement in conventional PM systems is not uncommon and occurs in 1.6% of all PM patients on average [21].

The concept of a leadless pacemaker (LP) may overcome all these issues mentioned above. As early as in the late 1960s, Dr. J. William Spickler and his colleagues were able to demonstrate the feasibility of a self-contained cardiac PM: Specifically, a canine with an iatrogenic heart block was paced with such a device for more than 2 months [22]. This technique, however, was initially limited by its short battery life. Meanwhile, battery technology improved substantially, the devices were further miniaturized, and the endocardial fixation mechanisms as well as the delivery systems could be enhanced. In December 2012, a new field of activity arose, when a LP (Nanostim Inc., Sunnyvale, CA, USA) was implanted for the first time in a human patient [23]. Currently, clinical data of two cardiac LP systems are available: Nanostim^®^ leadless cardiac pacemaker (LCP; Abbott Inc., Abbott Park, IL, USA) and Micra^®^ transcatheter pacing system (TPS; Medtronic Inc., Minneapolis, MN, USA). Both systems are fully self-contained cardiac PMs providing single-chamber right ventricular stimulation and carry specific sensors to enable rate-responsive pacing. Depending on programming and pacing indication, battery longevity is estimated up to 12 years. The implantation is performed in the cardiac catheterization laboratory or a (hybrid) operating room, where each device is delivered through the femoral vein and implanted into the right ventricle using a steerable catheter delivery system.

### Nanostim® leadless cardiac pacemaker (LCP)

The Nanostim^®^ LCP measures 41.4 × 6.0 mm and has a volume of 1 cm^3^. The device can be implanted through an 18F sheath (21F outer diameter), the fixation mechanism is a screw-in helix. In the LEADLESS trial, clinical performance and safety were tested in a “first-in-man” study of LP therapy [23]. This prospective trial enrolled 33 patients; the first implant was performed in December 2012 in Prague, Czech Republic. The overall implant success rate was as high as 97% (32/33), but acute repositioning of the system was required in 30% of patients (10/33). The overall complication-free survival at 3 months was 94% (31/33). One patient developed cardiac tamponade, which had to be repaired surgically and died 18 days after the intervention due to a cerebral ischemic infarct. In another patient, the device was accidentally implanted in the left ventricle via a patent foramen ovale but could be removed without any problems. At 1-year follow-up, there were no further adverse events [24]. The electrical performance measures of the device remained stable at 1 and 3 years of follow-up [24, 25]. The authors concluded that leadless pacing is feasible and safe and could represent a paradigm shift in cardiac pacing.

The following study (LEADLESS II trial) included 526 patients with an indication for permanent right ventricular (VVI) pacing [26]. The device was successfully implanted in 96% of patients; acute repositioning was necessary in 30%. During a follow-up of 6 months, major complications occurred in 6.5% of all patients, including cardiac perforation in 8 (6 of them required intervention), vascular complications in 6 and device dislodgement in further 6 cases. All dislodged devices were retrieved percutaneously. No device-related deaths occurred during the follow-up period. However, 2 mortalities during follow-up were classified procedure related (1 access complication with infection and 1 cerebral infarct after pericardiocentesis). Subsequently, implantations were temporarily stopped due to safety concerns, and an additional training program for implanting physicians was implemented. After the implementation of protocol improvements following the interruption of the study, the study’s primary safety endpoint was met and cardiac perforations and device dislodgements declined [27]. Recently, a premature battery failure was diagnosed in 34 out of 1423 implanted LCP devices, which was higher in number than initially expected. It occurred approximately 2.9 years after implantation [28]. In 7 patients, the device could not be interrogated any longer, and the battery depletion caused loss of pacing. As a consequence, the Nanostim^®^ LCP was transiently withdrawn from the market and is currently not commercially available.

### Micra® transcatheter pacing system (TPS)

**Fig. 1:**
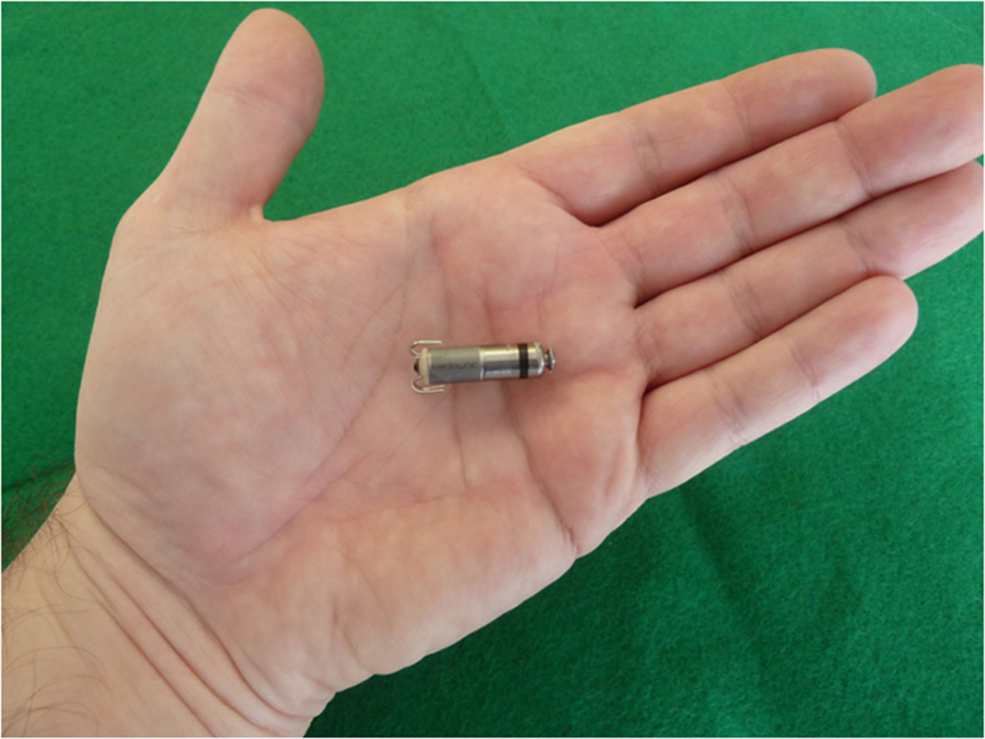
The Micra^®^ TPS LP system is currently the only available leadless pacing system. The first implantation in a human patient was performed in December 2013 in Linz, Austria

**Fig. 2:**
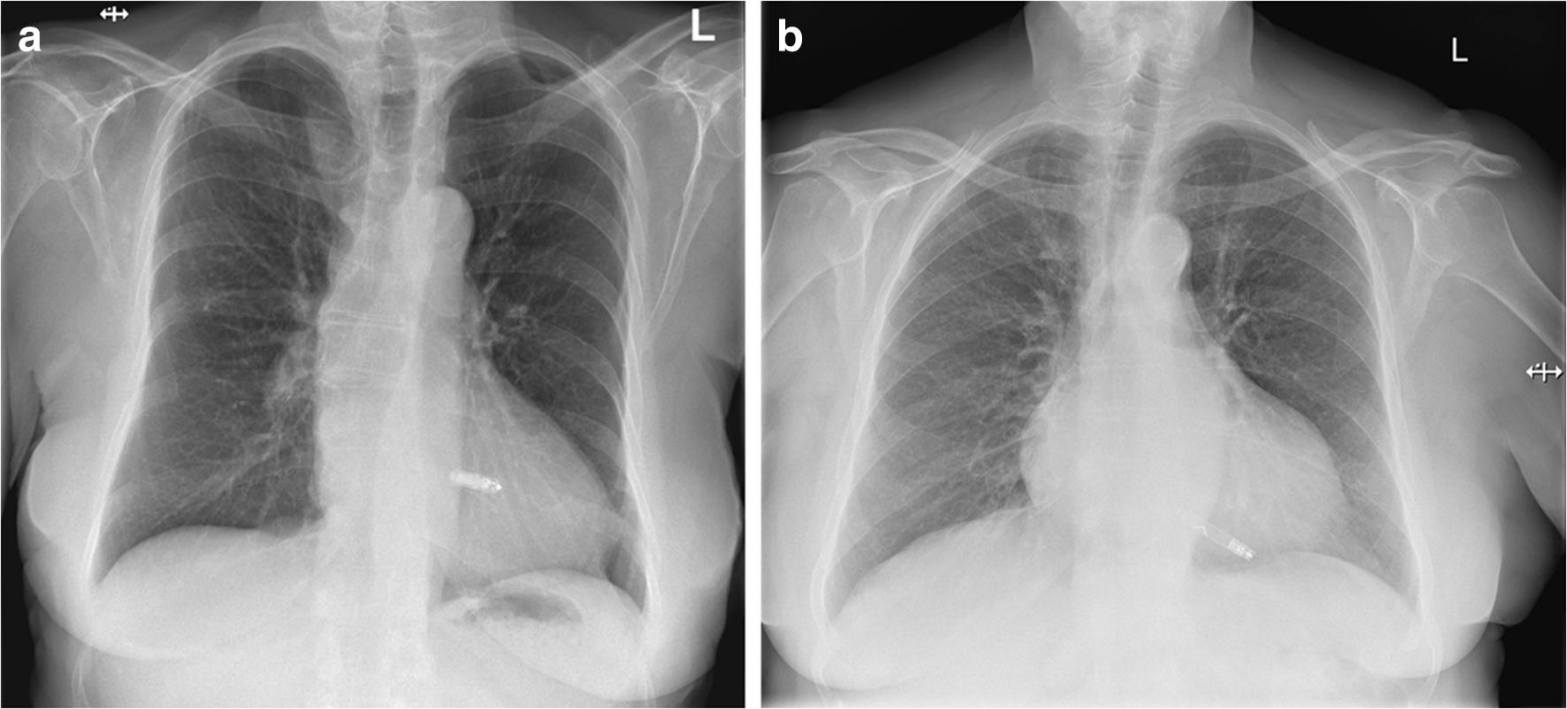
X-ray pictures of an implanted Micra^®^ TPS LP (a) and a Nanostim^®^ LCP system (b) are shown. The authors thank Prof. Dr. W. Jung, Schwarzwald-Baar Klinikum, Villingen-Schwenningen, Germany, for providing material for Fig. 2b

The Micra^®^ TPS is 26 mm in length, 7 mm in width, and 0.8 cm^3^ in volume (Fig. 1). Compared to the Nanostim^®^ LCP, this device is considerably shorter (difference 15 mm) but approximately 1 mm thicker, therefore requiring a 23F sheath (27F outer diameter) for implantation. The fixation mechanism is significantly different, using 4 self-expanding flexible nitinol tines. In December 2013, the first-in-human implantation of this device was performed at the Kepler University Hospital in Linz, Austria (Fig. 2).

Subsequently, safety and efficacy of this device was evaluated in a prospective study (Micra Transcatheter Pacing Study) [29, 30]. A total of 725 patients were included; implantation success rate was 99.2%; 719/725 patients). Overall, 28 major complications occurred in 25 patients (3.5%), including cardiac perforation or effusion in 11 and vascular complications in 5 patients. There were no radiographically visible device dislodgements during the follow-up period. One patient with end-stage renal failure died early after implantation. The cause of death was most likely a metabolic acidosis due to the prolonged procedure time. In 3 patients, system revisions were necessary (elevated pacing threshold, intermitted loss of capture, PM syndrome). At 12-months of follow-up, 32 major complications occurred cumulatively (24 complications within 30 days, 6 complications between 30 days and 6 months, and 2 complications after 6 months) [31]. Cardiac failure was the most frequent adverse event (6/8) after 30 days; however, no device-related death occurred during follow-up. Most importantly, no device dislodgements or infections were observed, and electrical performance measures remained stable during 12-months of follow-up.

The Micra Post-Approval Registry (PAR) was initiated to further evaluate the safety of the Micra^®^ TPS system prospectively in a “real-world” setting. Enrolment started in 2015 and the last patient was included in March 2018. In total, 1817 patients were enrolled. A interims analysis of safety and effectiveness data was published with 1 month [32] as well as 12 months of follow-up [33]. Interestingly, the “real-world” data were quite similar or even tended to be better than the results of the Micra Transcatheter Pacing Study: The implantation success rate remained high (99.1%), whereas the major complication rate was only 2.7% (41 patients). Pericardial effusion occurred in 14 patients, requiring pericardiocentesis in 8 and surgical repair in 2 of them. Both perforations with need for surgery ultimately led to death. There were another 11 patients with groin complications and one patient in whom the device dislodged with embolization and could be successfully repositioned 50 days after implantation. The authors concluded that the results demonstrate consistency with the results of the Micra Transcatheter Pacing Study and highlight the advantages of a LP in reducing complications associated with the components of conventional pacemakers.

### Comparison of leadless versus transvenous PM systems

There are currently no randomized controlled trials planned or published to compare efficacy and long-term safety between conventional transvenous PMs and LP systems. However, complications occur in up to 6.5% of LP implantations, which compares favorably to recently published complication rates of transvenous PM implantations. In the nationwide Danish cohort study, for instance, the complication rate was 6.9% for single-chamber systems [5].

The Micra Transcatheter Pacing Study compared the Micra^®^ TPS results with a historical control group comprising 2667 patients from 6 trials who had undergone transvenous PM implantation. At 12-months of follow-up, total major complications were significantly lower in the LP cohort (4.0% vs. 7.6%), resulting in a 48% relative risk reduction [31]. The specific subset of complications, however, varied in the two groups. The LP implantation has a unique risk of femoral vein complications, but no risk of pneumothorax or pocket problems. Moreover, more cardiac perforations are observed during LP implantations: In a meta-analysis of 28 studies, the mean incidence was about twice as high with LPs as compared with conventional PMs (conventional PMs 0.8% vs. 1.5% in the LEADLESS II and Micra Transcatheter Pacing Study [34]). However, it should be emphasized that the risk of perforation seems to be lower in the Micra TPS^®^ than in the Nanostim^®^ LCP and that damage to the RV can generally be avoided by aiming a more septal rather than apical LP position (Fig. 2). Indeed, the majority of Micra TPS^®^ were implanted in the RV septum at lower perforation rates in later studies [34]. In another report, data of all LP studies were compared with 10 studies with conventional transvenous PM implantations, including more than 14,000 patients [35]. Essentially, the short-term complication rate (< 2 months), mainly related to the implant procedure, was similar (4.8% in the LP group versus 4.1%). During long-term follow-up (> 2 months), the complication rate was considerably higher in the conventional PM group (3.1% versus 0.2%). Specifically, PM erosion or infection, pocket revision, or re-intervention due to lead failure or pacing threshold elevation only occurred in the conventional PM group.

There may be still unknown disadvantages of LP systems, such as the risk of “runaway pacing” due to electrical failure at the end of battery life, a complication that has been observed in earlier generations of transvenous PM. The latter or similar malfunctions have not been reported so far, but they could be managed by turning the LCP off by telemetry and therefore disconnecting the pulse generator from the circuit.

In summary, the advantages of LP could be the prevention of long-term complications, especially lead-related problems and pocket complications. However, further studies comparing leadless and conventional PMs on the long-term are needed to demonstrate the advantages of this new technology.

### Magnetic resonance imaging (MRI) compatibility of leadless pacemaker systems

As a large number of patients with cardiac implantable electronic devices (CIED) will also have to undergo an MRI scan, MRI compatibility of devices is an important issue. Both LP systems are MRI conditional and CE certified for full body MRI scans without an exclusion zone. In contrast, performance of MRI scans is not recommended during the lead maturation period (app. 6 weeks after implantation) of LP systems or in case of an elevated pacing threshold (> 4.5 V at the programmed pulse width), and MRI is contraindicated in the presence of concomitant devices or abandoned leads. Whereas the Micra^®^ TPS is approved for 1.5 T as well as 3.0 T scanners, the Nanostim^®^ LCP is approved for 1.5 T scanners only. Ex vivo as well as real-life data confirmed the safety and feasibility of MRI scans with good image quality of even cardiac MRIs [36–39]. Specifically, pacing thresholds (changes vs. baseline 0.01 ± 0.05 V) and other device parameters remained stable [40], and device dislodgements have not been observed to be increased by MRI scans in LP systems [41]. However, both LP systems need to be programmed to a specific MRI mode prior to the scan, according to the manufacturers’ recommendations and the ESC guidelines for cardiac pacing [1]. Thereby, pacing is turned off (0V0 mode) in patients without the need for pacing support, or an asynchronous (V00) mode is chosen with a pacing rate well above the patient’s intrinsic heart rate in patients, who are either PM dependent or in whom pacing support is desired for other reasons. After the scan, devices must be re-interrogated and original settings must be restored.

### Management of device infections: advantages of leadless pacers

Pocket- and lead-related complications are seen in 7–12% of patients with conventional PMs and lead to surgical interventions in about 4%, especially in the case of infection [42]. Infections of CIED inherit a high individual risk with a mortality rate of 20% within the first year after onset, a substantial risk of reinfection of more than 10%, and a significant healthcare burden due to the need for increased surgery utilization and a long hospital stay [14, 43, 44]. The incidence of major CIED infections can be reduced by the adjunctive use of an absorbable, antibiotic-eluting envelope (“TYRX^®^; Medtronic Inc.”). Indeed, a reduction of device infections from 1,2 to 0,7% has been observed by this simple but expensive tool in a randomized, controlled clinical trial [45]. LPs were designed to eliminate or reduce pocket- and lead-related complications, especially infection, and may provide a safe and attractive pacing option for patients with previous infection of a conventional PM.

Both, the “Micra investigational device exemption (IDE) study” with 726 patients and the “Micra Post-Approval Registry (PAR)” with 1817 patients and a current follow-up of up to 24 months, reported no infections of the Micra^®^ TPS requiring surgical or interventional treatment [31, 33]. It is of special interest that the majority of patients enrolled in both these studies exhibited established risk factors for CIED infection like diabetes mellitus, renal dysfunction or renal failure, anticoagulation, or chronic use of corticosteroids [46]. The same results, albeit with a smaller number of patients, also apply to the Nanostim^®^ LCP which is currently not commercially available [25].

In contrast to the IDE study, PAR also included patients with prior CIED explantation for infection. In these 99 patients (mean age 73 years), a Micra^®^ TPS could be successfully implanted in 99% after complete or partial explantation of the prior CIED (in 93% and 7%, respectively). The mean duration between the prior CIED explantation and the LP implantation was 6 days. Most of the patients could be discharged from the hospital 2 days after implantation (interquartile range: 1–7 days). At a mean follow-up of 6 months (range 0–25 months), no recurrent infections requiring removal of the Micra^®^ TPS occurred. The authors concluded that LPs appear to be a safe pacing alternative in patients with recent CIED explantation for severe infection [47].

A rationale for the prevention of device infection with LPs may be the material of the capsules and their comparatively small size. The surface of the LP capsules is made of pure titanium that is not altered by environmental influences of the surrounding tissue. In contrast, the much larger surface of the can and leads of conventional PMs is more prone to be colonized by bacteria. Especially the leads, covered by polyurethane or polydimethylsiloxane, degrade over time, giving rise to a pro-coagulatory and microbe-attracting milieu [48]. Another reason for the resilience against infection of LPs may be the observed early and complete encapsulation after implantation that forms an endothelialized capsule, which is able to prevent the LP from reinfection even in patients with previous severe CIED infection [49–52].

In summary, LPs reduce or even eliminate the risk of infection in both patients with multiple clinical risk factors for developing a first event and those after explantation of a CIED for severe infection. Thus, implantation of an LP could be an alternative to a “TYRX^®^” antibiotic-eluting envelope in patients after CIED infection unless they need atrioventricular pacing, cardiac resynchronization, or a transvenous ICD (Table 1). Furthermore, patients without previous CIED infection, who are suitable for a LP according to the underlying arrhythmia (see below), are possible candidates for LP implantation, if at least 2 of the following clinical risk factors for CIED infection are present:

- Diabetes mellitus
- Renal dysfunction
- Chronic use of corticosteroids
- Recurrent systemic infections or immunosuppressive therapy

**Table 1:** Recommendations for indications for leadless pacemaker (LP) therapy: risk of infections

Good candidate for LP implantation: - History of CIED infection - No need for AV sequential pacing, CRT or transvenous ICD Possible candidate for LP implantation: - Suitable for a LP according to underlying arrhythmia - Two or more clinical risk factors for device infection: diabetes mellitus, renal dysfunction or chronic hemodialysis, chronic use of corticosteroids, recurrent systemic infections, or immunosuppressive therapy	

## A national consensus for indications of leadless pacing in Austria

Given the characteristics and the specific implantation technique of LP systems as well as rapidly increasing implantation rates, it is mandatory to define indications for patients who might particularly benefit from these novel devices. Thus, an expert consensus was defined by a group of skilled PM implanters in Austria with experience in LP technology. The distinct mission of these arrhythmia specialists was to define a consensus on indications of LP based on current evidence.

- A “Good candidate for LP implantation” recommendation denotes scenarios in which patients clearly benefit from an LP as opposed to a conventional PM system according to published studies.
- A “Possible candidate for LP implantation” recommendation was issued, if the majority of the experts favored a LP system.
- A “Possible candidate for LP implantation under certain circumstances” recommendation was issued, if the indication was not deemed advantageous by the majority of the panel.
- A “No candidate for LP implantation” recommendation includes all current contraindications for LP therapy that may potentially harm the patient in these cases.

The recommendations were separately defined for the underlying arrhythmia and for special clinical circumstances and should help to decide between a conventional PM and a LP system. It has to be emphasized that no randomized controlled study has been conducted so far to compare conventional transvenous pacers and LP systems. Thus, these consensus recommendations are based on observational LP trials without control groups, comparisons of registry data with historic controls, and expert opinions. In order to highlight the scientific background for the readers, according to current guidelines, the level of evidence of the recommendations was defined as “B”, if published studies underlined the consensus, and as “C”, if the recommendation was solely based on opinion of the expert panel.^1^

### Pacing indications based on the underlying arrhythmia

Although the technology of LP is rapidly evolving, currently available devices are capable of ventricular sensing and pacing only. Thus, operating modes are limited to VVI and VVI-R [1]. Frequent (asynchronous) ventricular pacing of patients in sinus rhythm can lead to an increased rate of AF and stroke [53] as well as reduced quality of life due to a “pacemaker-syndrome” and reduced exercise performance [54] compared to dual-chamber pacing. The cutoff for ventricular pacing burden that might lead to clinically important adverse events is currently unclear, but AV synchrony needs to be preserved in patients with sinus rhythm as intensively as possible.

Thus, the panel recommends LP preferentially in patients with permanent AF and AV block or slow ventricular response. Patients in sinus rhythm with transient sinus arrest or AV block and a very low anticipated ventricular pacing rate (≤ 1–5% of beats) are similarly good candidates for LP implantation a LP system in case of difficult venous subclavian access, a history of infection, or risk of tricuspid valve dysfunction (Table 2). In contrast, transvenous dual-chamber PM systems should be preferred in patients with sick sinus syndrome, vasovagal syncope, or AV block with transient or permanent bradycardia. These patients are expected to be paced primarily in the atrium or frequently and AV sequentially in the ventricle (Table 2). Leadless or transvenous systems may equally be considered in inactive patients in sinus rhythm, since LP implantation can be performed safely and single-chamber pacing does not increase mortality or cause symptoms in this subgroup of patients [55]. Furthermore, the panel anticipates that indications may broaden quickly with the development LP systems capable of atrial and/or AV synchronous pacing underlining the need of re-evaluation of indications based on technical progress.

**Table 2:** Indications for leadless pacemaker (LP) therapy according to the underlying arrhythmia: clinical evidence is graded as A, B, or C; * good candidate for LP implantation (evidence B) in elderly, inactive patients

Good candidate for LP implantation (evidence B): • Permanent AF and AV block or slow ventricular response Possible candidate for LP implantation (evidence B): • Transient sinus arrest or AV block with need of backup pacing and very low anticipated ventricular pacing rate (≤ 1–5% of beats)	
Possible candidate for LP implantation under certain circumstances (evidence C): • Transient or permanent AV block with increased anticipated ventricular pacing rate (> 1–5% of beats) • Sick sinus syndrome with transient or permanent bradycardia with increased anticipated ventricular pacing rate (> 1–5% of beats)* • Recurrent syncope due to vagally induced cardio-inhibition (sinus bradycardia or transient AV block)

**Table 3:** Indications for leadless pacemaker (LP) therapy according to clinical circumstances: clinical evidence is graded as A, B, or C

Good candidate for LP implantation (evidence C): • Missing or difficult venous subclavian access • History of complications of PM therapy (especially infection) • Elevated risk of complications or risk of tricuspid valve dysfunction (e.g., severe TV regurgitation or reconstructed TV) Possible candidate for LP implantation (evidence C): • Frequent sports activity stressing the shoulders (golf, hunting, etc.) • Age < 65 years • Children and adolescents < 20 years of age No candidate for LP implantation (evidence C): • Expected high ventricular pacing burden and moderate to severe LV dysfunction (LVEF ≤ 35%)

**Table 4:** Qualification and technical requirements for implantation of LP systems: clinical evidence is graded as A, B, or C

LP implantation should be performed in (evidence C): • A catheterization laboratory or hybrid surgery room equipped with a high-resolution fluoroscopy system • An implanting center with experience and equipment for instant pericardiocentesis and/or surgical defect closure, fast access to a surgery department with the availability of cardiopulmonary bypass (transport time < 1 h) LP implantation should be performed by (evidence C): • An implanter with experience in venous femoral access • An implanter with experience in manipulation of sheaths in the right atrium and ventricle • An implanter certified by the manufacturer (proctoring by experienced LP implanter for the first 3 cases recommended) LP implantation should be considered to be performed in (evidence C): • An implanting center with experience in surgical and interventional device extraction • Implanting center with experience in surgical and interventional management of vascular complications	

### Clinical circumstances that favor either LP or transvenous PM

In addition to the underlying arrhythmia, there are several clinical circumstances and comorbidities that influence the decision between use of an LP and a conventional transvenous PM system: According to the manufacturer’s recommendations, implantation of an LP is contraindicated in patients with mechanical tricuspid valves (as are conventional transvenous leads), since the delivery system and/or the pacing device can be trapped and the valve can be damaged during the implant procedure. In the case of bilaterally occluded subclavian veins, a superior V. cava syndrome, or various forms of congenital heart disease, the right ventricle can only be accessed intravenously via the femoral vein and the inferior V. cava. Thus, utilization of an LP system is recommended in these rare cases. Furthermore, a LP system, as opposed to a transvenous or an epicardial PM system, should explicitly be preferred in patients with a history of severe PM system infection and in patients with dysfunctional or reconstructed tricuspid valves or after biological tricuspid valve replacement, since these patients are prone to recurrent infections as well as the development or aggravation of tricuspid valve dysfunction (Table 3). Preferential use of an LP system may also be considered in patients with an expected elevated risk of complications (hemodialysis, advanced pulmonary disease, very low body mass index, frailty), since retrospective data indicate a favorable complication rate in these high-risk patients during midterm follow-up [31, 56]. However, patients with risk factors mentioned above also have an elevated risk of complications with LP systems, and this recommendation is limited by the absence of prospective, randomized data. Thus, decision to implant an LP should still be based on weighting potential benefits and risks in patients at risk for complications mentioned above.

According to recent registry data and extrapolation of battery drainage measurements, life span of LP systems is estimated to be 7–10 years [57]. Thereafter, there are basically two options: The LP can either be extracted [58] and replaced by a new (leadless or transvenous) system, or another LP can be implanted right next to the previous one into the right ventricle [59]. At the moment, it is unclear which of these options shall be preferred, as data from larger trials are still missing. Thus, end-of-life management in LP patients currently remains an open discussion. As benefits of LP systems may be counterbalanced by complications of extraction procedures and interference of multiple intracardiac devices, the panel does not recommend LP implantations in children/adolescents below 20 years of age or in patients who prefer these systems just for cosmetic reasons or due to convenience issues. Given a median life expectancy of 80 years of the general population in Central Europe and an estimated LP system durability of about 10 years, more than two LP systems may need to be implanted in patients with pacing indication below the age of 65 years. Because of the uncertainties of LP end-of-life management, conventional transvenous pacing systems should be preferred in patients < 65 years of age. By contrast PM patients exposed to certain professional or athletic activities (e.g., golf, hunting, scuba diving) [60] might benefit from a lower complication rate of LP systems positioned directly within the heart. However, the final decision which pacing system to implant has to be made individually in these cases, based on a careful risk-benefit calculation.

Finally, a transvenous or epicardial biventricular system should be preferred in patients with a pacing indication and anticipated high ventricular pacing rate, heart failure, and impaired left ventricular function (LVEF ≤ 35%). In these patients, biventricular pacing systems should be preferred to enable cardiac resynchronization therapy [1]. An LP or alternatively an epicardial PM system may only be considered in these particular cases, if transvenous subclavian access to the right ventricle and/or to the coronary sinus is impossible.

In conclusion, patients with missing or difficult bilateral subclavian or superior V. cava access, as well as patients with a history of complications, who need a single-chamber pacing system, are good LP candidates. However, potential benefits of this novel therapy such as the reduced risk of complications must be weighed against the lack of long-term experience and yet undetermined end-of-life management. Therefore, all issues mentioned above need to be discussed within the implanting team and – even more importantly – with the patients themselves, especially in borderline situations ("possible candidate").

## Standards for implantation and surgical management of leadless pacing

As outlined above, most complications in LP occur within the first 2 months of therapy and are primarily related to the implantation procedure itself. Specifically, acute vascular injury, perforation and cardiac tamponade, as well as extraction of embolized or malfunctioning systems need to be managed by the treating physicians and their team. Thus, the expert panel defined technical requirements for LP implantation and medical qualification of the implanting physicians in Austria.

### Qualification and technical standards for implantation

The panel defined specific standards for implantation to provide an optimal setting for this novel technology. Due to the specific implantation technique, a very low risk of perioperative infection is supposed. However, the high standards for conventional PM implantation regarding air quality and sterile technique should also be maintained for this group of devices. In addition, a high-resolution X-ray system is of absolute importance for the currently available transcatheter LP system. A conventional C-arm is not sufficient to test movement and optimal placement of tines or to observe micro-dislodgement of the device. Thus, we recommend that LP implantations are performed either in a hybrid operating room or a catheterization lab equipped with an air piping system (Table 4). Furthermore, hygienic conditions and functional specifications have to be organized as with any other CIED implantation to avoid device and access site infections. Availability of an on-site cardiac and/or vascular surgery team may be life-saving in case of urgent vascular complications, embolization of the device, or ventricular perforation. Thus, the panel recommends that LP systems should only be implanted in hospitals prepared to manage these types of emergencies. Specifically, while on-site open-heart surgery is not a prerequisite of LP implantation centers, close cooperation and fast access to emergency surgical support close to the site are required. LP implantations can be safely performed under local anesthesia or conscious sedation; however, under certain circumstances, general anesthesia may be required.

LP implanters have to be experienced in femoral venous punctures and the handling with large sheaths (above 11 French). Moreover, the procedure should be performed by cardiac electrophysiologists, interventional cardiologists, or surgeons with experience in transvenous PM implantations and/or electrophysiological procedures in the right atrium and ventricle according to our recommendation. A specific training is of utmost importance to provide safety and ensure procedural success. Currently, this training consists of two consecutive parts. First, the different procedural steps are presented online in an e-learning program (provided by the manufacturer), which has to be completed before starting the hands-on training in experienced centers. After this training, the first at least 3–5 implantations should be supported by a certified proctor in order to assure a step-by-step guidance to avoid adverse outcomes. When launching a new LP program, we recommend taking advantage of the described LP implant training modules. However, a retraining after several successful implantations is not deemed necessary by the panel. Finally, due to the steep learning curve in LP implantation procedures, we did not define a minimal number of LP implantation procedures per operator within a certain time frame. However, as in all interventional procedures, all operators should aim for a proper case load from the very beginning of an institution’s LP program.

### Surgical management

#### Groin and vascular injury

The venous puncture site may be closed by heavy silk sutures (“Z” suture, purse string sutures [61]) or by a Perclose Proglide^®^ system (Abbott Inc., USA). Additionally, a compression dressing should be applied after implantation for at least 6 h in the case of access site bleeding or if immediate hemostasis cannot be achieved. Injuries of the venous system are rare and may be prevented by ultrasound-guided vascular access and venography given the large sheath size of the catheters. In the majority of cases, surgical management is not required; however, accidental arterial injury may lead to scenarios involving vascular surgery or interventional management. The panel therefore recommends vascular imaging during puncture and introduction of the sheaths as well as close cooperation of the implanting team with a cardiac and/or vascular surgery department to be able to immediately manage these complications as described above.

#### Pericardial tamponade

Since the risk of perforation is elevated, if LP is implanted in the apex rather than the septum of the RV, latter position of LP should be preferred. Furthermore, blood pressure needs to be monitored invasively or noninvasively during the entire procedure to identify bleeding events as soon as possible. Any new-onset pericardial effusion, even if not hemodynamically relevant, can instantly be visualized by echocardiography. If hemodynamics are compromised, interventional pericardial drainage or surgical defect closure needs to be performed immediately. Thus, fast access to equipment and experience of the implanting team in emergency pericardiocentesis are required. Although cardiac tamponade is very rare, it may exhibit a more rapid onset compared to conventional PM implantations due to the larger sheath size. Thus, fast access to a surgery department with the availability of cardiopulmonary bypass (transport time < 1 h) is required according to the recommendation of this expert panel.

#### System retrieval

The need for system retrieval is expected to be rare and is currently limited to specific issues, like endocarditis or system upgrades [62, 63]. Especially, “late” extractions after more than 3 months have been rarely reported so far [62]. Thus, we do not recommend routine system extraction, if the battery’s “elective replacement indicator” is attained. Additional LP devices can probably be implanted next to earlier implants without extraction of previous devices (in this case, they have to be programmed to the device off [*0V0*] mode), but current clinical experience is very limited. Interestingly, a postmortem series showed that up to three Micra^®^ devices were implantable in the human right ventricle [59] without interacting mechanically or electronically with one another.

If a strong indication for system extraction is present, it might be feasible with a LP delivery sheath or a deflectable sheath introduced via femoral access and a snare catheter during the 1st year after implantation and in infected devices. However, adhesions and tissue overgrowth may be a limiting factor with a dwell time of more than 1 year. Since there is an elevated risk of perforation and tamponade in the case of extraction of any chronically implanted CIED, these procedures should preferentially be performed in a hybrid operating room setting with cardiopulmonary bypass standby [64].

## Conclusion

This article gives an overview of LP systems and summarizes recommendations of an expert panel comprising a number of PM implanters in Austria with experience in LP technology. The authors give a short overview about the current evidence of LP but also define suggested indications, management of PM infections, and advice for the implantation procedure.

The published recommendations reflect the views of the authors and of the working group of arrhythmias of the Austrian Society of Cardiology (ÖKG), and it is emphasized that they do not necessarily represent the views of other national or international societies.

## References

[1] Brignole M, Auricchio A, Baron-Esquivias G, Bordachar P, Boriani G, Breithardt OA, Cleland J, Deharo JC, Delgado V, Elliott PM, Gorenek B, Israel CW, Leclercq C, Linde C, Mont L, Padeletti L, Sutton R, Vardas PE, Zamorano JL, Achenbach S, Baumgartner H, Bax JJ, Bueno H, Dean V, Deaton C, Erol C, Fagard R, Ferrari R, Hasdai D, Hoes AW, Kirchhof P, Knuuti J, Kolh P, Lancellotti P, Linhart A, Nihoyannopoulos P, Piepoli MF, Ponikowski P, Sirnes PA, Tamargo JL, Tendera M, Torbicki A, Wijns W, Windecker S, Kirchhof P, Blomstrom-Lundqvist C, Badano LP, Aliyev F, Bänsch D, Baumgartner H, Bsata W, Buser P, Charron P, Daubert JC, Dobreanu D, Faerestrand S, Hasdai D, Hoes AW, le Heuzey JY, Mavrakis H, McDonagh T, Merino JL, Nawar MM, Nielsen JC, Pieske B, Poposka L, Ruschitzka F, Tendera M, van Gelder I, Wilson CM, ESC Committee for Practice Guidelines (CPG), Document Reviewers (2013). 2013 ESC guidelines on cardiac pacing and cardiac resynchronization therapy: the task force on cardiac pacing and resynchronization therapy of the European Society of Cardiology (ESC). Developed in collaboration with the European heart rhythm association (EHRA). Eur Heart J.

[2] Epstein AE, DiMarco JP, Ellenbogen KA, Estes NA, Freedman RA, Gettes LS (2013). 2012 ACCF/AHA/HRS focused update incorporated into the ACCF/AHA/HRS 2008 guidelines for device-based therapy of cardiac rhythm abnormalities: a report of the American College of Cardiology Foundation/American Heart Association task force on practice guidelines and the Heart Rhythm Society. J Am Coll Cardiol.

[3] Larsson B, Elmqvist H, Ryden L, Schuller H (2003). Lessons from the first patient with an implanted pacemaker: 1958-2001. Pacing Clin Electrophysiol.

[4] Mond HG, Proclemer A (2011). The 11th world survey of cardiac pacing and implantable cardioverter-defibrillators: calendar year 2009--a world society of Arrhythmia's project. Pacing Clin Electrophysiol.

[5] Kirkfeldt RE, Johansen JB, Nohr EA, Jorgensen OD, Nielsen JC (2014). Complications after cardiac implantable electronic device implantations: an analysis of a complete, nationwide cohort in Denmark. Eur Heart J.

[6] Udo EO, van Hemel NM, Zuithoff NP, Barrett MJ, Ruiter JH, Doevendans PA (2013). Incidence and predictors of pacemaker reprogramming: potential consequences for remote follow-up. Europace..

[7] Lin G, Nishimura RA, Connolly HM, Dearani JA, Sundt TM, Hayes DL (2005). Severe symptomatic tricuspid valve regurgitation due to permanent pacemaker or implantable cardioverter-defibrillator leads. J Am Coll Cardiol.

[8] Delling FN, Hassan ZK, Piatkowski G, Tsao CW, Rajabali A, Markson LJ, Zimetbaum PJ, Manning WJ, Chang JD, Mukamal KJ (2016). Tricuspid regurgitation and mortality in patients with transvenous permanent pacemaker leads. Am J Cardiol.

[9] Haghjoo M, Nikoo MH, Fazelifar AF, Alizadeh A, Emkanjoo Z, Sadr-Ameli MA (2007). Predictors of venous obstruction following pacemaker or implantable cardioverter-defibrillator implantation: a contrast venographic study on 100 patients admitted for generator change, lead revision, or device upgrade. Europace..

[10] Santini M, Di Fusco SA, Santini A, Magris B, Pignalberi C, Aquilani S (2016). Prevalence and predictor factors of severe venous obstruction after cardiovascular electronic device implantation. Europace..

[11] Johansen JB, Jorgensen OD, Moller M, Arnsbo P, Mortensen PT, Nielsen JC (2011). Infection after pacemaker implantation: infection rates and risk factors associated with infection in a population-based cohort study of 46299 consecutive patients. Eur Heart J.

[12] Klug D, Balde M, Pavin D, Hidden-Lucet F, Clementy J, Sadoul N (2007). Risk factors related to infections of implanted pacemakers and cardioverter-defibrillators: results of a large prospective study. Circulation..

[13] Brunner MP, Cronin EM, Duarte VE, Yu C, Tarakji KG, Martin DO, Callahan T, Cantillon DJ, Niebauer MJ, Saliba WI, Kanj M, Wazni O, Baranowski B, Wilkoff BL (2014). Clinical predictors of adverse patient outcomes in an experience of more than 5000 chronic endovascular pacemaker and defibrillator lead extractions. Heart Rhythm.

[14] Tarakji KG, Wazni OM, Harb S, Hsu A, Saliba W, Wilkoff BL (2014). Risk factors for 1-year mortality among patients with cardiac implantable electronic device infection undergoing transvenous lead extraction: the impact of the infection type and the presence of vegetation on survival. Europace..

[15] Gomes S, Cranney G, Bennett M, Giles R (2016). Long-term outcomes following transvenous lead extraction. Pacing Clin Electrophysiol.

[16] Athan E, Chu VH, Tattevin P, Selton-Suty C, Jones P, Naber C (2012). Clinical characteristics and outcome of infective endocarditis involving implantable cardiac devices. JAMA..

[17] Benz AP, Vamos M, Erath JW, Hohnloser SH. Cephalic vs subclavian lead implantation in cardiac implantable electronic devices: a systematic review and meta-analysis. Europace. 2018. 10.1093/europace/euy165. 10.1093/europace/euy165 30020452

[18] de Vries LM, Leening MJG, Dijk WA, Hooijschuur CAM, Stricker BH, van Hemel NM. Trends in replacement of pacemaker leads in the Netherlands: a long-term nationwide follow-up study. Pacing Clin Electrophysiol. 2018. 10.1111/pace.13371. 10.1111/pace.13371 29749035

[19] Hauser RG, Hayes DL, Kallinen LM, Cannom DS, Epstein AE, Almquist AK, Song SL, Tyers GF, Vlay SC, Irwin M (2007). Clinical experience with pacemaker pulse generators and transvenous leads: an 8-year prospective multicenter study. Heart Rhythm.

[20] Birgersdotter-Green UM, Pretorius VG (2014). Lead extractions: indications, procedural aspects, and outcomes. Cardiol Clin.

[21] Wang Y, Hou W, Zhou C, Yin Y, Lu S, Liu G, et al. Meta-analysis of the incidence of lead dislodgement with conventional and leadless pacemaker systems. Pacing Clin Electrophysiol. 2018. 10.1111/pace.13458. 10.1111/pace.13458 30066363

[22] Spickler JW, Rasor NS, Kezdi P, Misra SN, Robins KE, LeBoeuf C (1970). Totally self-contained intracardiac pacemaker. J Electrocardiol.

[23] Reddy VY, Knops RE, Sperzel J, Miller MA, Petru J, Simon J, Sediva L, de Groot JR, Tjong FV, Jacobson P, Ostrosff A, Dukkipati SR, Koruth JS, Wilde AA, Kautzner J, Neuzil P (2014). Permanent leadless cardiac pacing: results of the LEADLESS trial. Circulation.

[24] Knops RE, Tjong FV, Neuzil P, Sperzel J, Miller MA, Petru J (2015). Chronic performance of a leadless cardiac pacemaker: 1-year follow-up of the LEADLESS trial. J Am Coll Cardiol.

[25] Tjong FVY, Knops RE, Neuzil P, Petru J, Sediva L, Wilde AAM, Sperzel J, Reddy VY (2018). Midterm safety and performance of a leadless cardiac pacemaker: 3-year follow-up to the LEADLESS trial (Nanostim safety and performance trial for a leadless cardiac pacemaker system). Circulation..

[26] Reddy VY, Exner DV, Cantillon DJ, Doshi R, Bunch TJ, Tomassoni GF (2015). Percutaneous implantation of an entirely Intracardiac leadless pacemaker. N Engl J Med.

[27] Sperzel J, Defaye P, Delnoy PP, Garcia Guerrero JJ, Knops RE, Tondo C, Deharo JC, Wong T, Neuzil P (2018). Primary safety results from the LEADLESS observational study. Europace..

[28] Lakkireddy D, Knops R, Atwater B, Neuzil P, Ip J, Gonzalez E (2017). A worldwide experience of the management of battery failures and chronic device retrieval of the Nanostim leadless pacemaker. Heart Rhythm.

[29] Reynolds D, Duray GZ, Omar R, Soejima K, Neuzil P, Zhang S, Narasimhan C, Steinwender C, Brugada J, Lloyd M, Roberts PR, Sagi V, Hummel J, Bongiorni MG, Knops RE, Ellis CR, Gornick CC, Bernabei MA, Laager V, Stromberg K, Williams ER, Hudnall JH, Ritter P, Micra Transcatheter Pacing Study Group (2016). A leadless intracardiac transcatheter pacing system. N Engl J Med.

[30] Ritter P, Duray GZ, Steinwender C, Soejima K, Omar R, Mont L (2015). Early performance of a miniaturized leadless cardiac pacemaker: the Micra Transcatheter Pacing Study. Eur Heart J.

[31] Duray GZ, Ritter P, El-Chami M, Narasimhan C, Omar R, Tolosana JM (2017). Long-term performance of a transcatheter pacing system: 12-month results from the Micra Transcatheter Pacing Study. Heart Rhythm.

[32] Roberts PR, Clementy N, Al Samadi F, Garweg C, Martinez-Sande JL, Iacopino S (2017). A leadless pacemaker in the real-world setting: the Micra Transcatheter Pacing System post-approval registry. Heart Rhythm.

[33] El-Chami MF, Al-Samadi F, Clementy N, Garweg C, Martinez-Sande JL, Piccini JP (2018). Updated performance of the Micra transcatheter pacemaker in the real-world setting: a comparison to the investigational study and a transvenous historical control. Heart Rhythm.

[34] Vamos M, Erath JW, Benz AP, Bari Z, Duray GZ, Hohnloser SH (2017). Incidence of cardiac perforation with conventional and with leadless pacemaker systems: a systematic review and meta-analysis. J Cardiovasc Electrophysiol.

[35] Tjong FV, Reddy VY (2017). Permanent leadless cardiac pacemaker therapy: a comprehensive review. Circulation.

[36] Soejima K, Edmonson J, Ellingson ML, Herberg B, Wiklund C, Zhao J (2016). Safety evaluation of a leadless transcatheter pacemaker for magnetic resonance imaging use. Heart Rhythm.

[37] Blessberger H, Kiblboeck D, Reiter C, Lambert T, Kellermair J, Schmit P, et al. Monocenter Investigation Micra(R) MRI study (MIMICRY): feasibility study of the magnetic resonance imaging compatibility of a leadless pacemaker system. Europace. 2018. 10.1093/europace/euy143. 10.1093/europace/euy143 29986008

[38] Kiblboeck D, Reiter C, Kammler J, Schmit P, Blessberger H, Kellermair J (2018). Artefacts in 1.5 Tesla and 3 Tesla cardiovascular magnetic resonance imaging in patients with leadless cardiac pacemakers. J Cardiovasc Magn Reson.

[39] Lobe S, Hilbert S, Hindricks G, Jahnke C, Paetsch I (2018). Cardiovascular magnetic resonance imaging in a patient with implanted leadless pacemaker. JACC Clin Electrophysiol.

[40] Blessberger H, Kiblboeck D, Reiter C, Lambert T, Kellermair J, Schmit P (2019). Monocenter Investigation Micra(R) MRI study (MIMICRY): feasibility study of the magnetic resonance imaging compatibility of a leadless pacemaker system. Europace.

[41] Wang Y, Hou W, Zhou C, Yin Y, Lu S, Liu G, Duan C, Cao M, Li M, Toft ES, Zhang HJ (2018). Meta-analysis of the incidence of lead dislodgement with conventional and leadless pacemaker systems. Pacing Clin Electrophysiol.

[42] Udo EO, Zuithoff NP, van Hemel NM, de Cock CC, Hendriks T, Doevendans PA, Moons KG (2012). Incidence and predictors of short- and long-term complications in pacemaker therapy: the FOLLOWPACE study. Heart Rhythm.

[43] Sohail MR, Henrikson CA, Braid-Forbes MJ, Forbes KF, Lerner DJ (2011). Mortality and cost associated with cardiovascular implantable electronic device infections. Arch Intern Med.

[44] Boyle TA, Uslan DZ, Prutkin JM, Greenspon AJ, Baddour LM, Danik SB, et al. Reimplantation and repeat infection after cardiac-implantable electronic device infections: experience from the MEDIC (Multicenter Electrophysiologic Device Infection Cohort) database. Circ Arrhythm Electrophysiol. 2017;10(3). 10.1161/CIRCEP.116.004822. 10.1161/CIRCEP.116.004822 28292753

[45] Tarakji KG, Mittal S, Kennergren C, Corey R, Poole JE, Schloss E (2019). Antibacterial envelope to prevent cardiac implantable device infection. N Engl J Med.

[46] Bloom H, Heeke B, Leon A, Mera F, Delurgio D, Beshai J, Langberg J (2006). Renal insufficiency and the risk of infection from pacemaker or defibrillator surgery. Pacing Clin Electrophysiol.

[47] JJ E-CMF, Zaidi A, Faerestrand S, Reynolds D. Garcia-Seara J Leadless pacemaker implant in patients with pre-existing infections: results from the Micra post-approval registry. Heart rhythm scientific sessions, Boston. May 10 2018:MA2018.

[48] Tohfafarosh M, Sevit A, Patel J, Kiel JW, Greenspon A, Prutkin JM, Kurtz SM (2016). Characterization of outer insulation in long-term-implanted leads. J Long-Term Eff Med Implants.

[49] Kypta A, Blessberger H, Kammler J, Lichtenauer M, Lambert T, Silye R et al. First autopsy description of changes 1 year after implantation of a leadless cardiac pacemaker: unexpected ingrowth and severe chronic inflammation. Can J Cardiol. 2016;32(12):1578 e1- e2. doi:10.1016/j.cjca.2015.12.028. 10.1016/j.cjca.2015.12.028 26927858

[50] Kypta A, Blessberger H, Lichtenauer M, Steinwender C (2016). Complete encapsulation of a leadless cardiac pacemaker. Clin Res Cardiol.

[51] Kypta A, Blessberger H, Kammler J, Lambert T, Lichtenauer M, Brandstaetter W, Gabriel M, Steinwender C (2016). Leadless cardiac pacemaker implantation after lead extraction in patients with severe device infection. J Cardiovasc Electrophysiol.

[52] Vamos M, Honold J, Duray GZ, Hohnloser SH (2016). MICRA leadless pacemaker on autopsy. JACC Clin Electrophysiol.

[53] Nielsen JC, Thomsen PE, Hojberg S, Moller M, Vesterlund T, Dalsgaard D (2011). A comparison of single-lead atrial pacing with dual-chamber pacing in sick sinus syndrome. Eur Heart J.

[54] Lamas GA, Lee KL, Sweeney MO, Silverman R, Leon A, Yee R, Marinchak RA, Flaker G, Schron E, Orav EJ, Hellkamp AS, Greer S, McAnulty J, Ellenbogen K, Ehlert F, Freedman RA, Estes NA, Greenspon A, Goldman L, Mode Selection Trial in Sinus-Node Dysfunction (2002). Ventricular pacing or dual-chamber pacing for sinus-node dysfunction. N Engl J Med.

[55] Lamas GA, Orav EJ, Stambler BS, Ellenbogen KA, Sgarbossa EB, Huang SK (1998). Quality of life and clinical outcomes in elderly patients treated with ventricular pacing as compared with dual-chamber pacing. Pacemaker selection in the elderly investigators. N Engl J Med.

[56] Tjong FVY, Knops RE, Udo EO, Brouwer TF, Dukkipati SR, Koruth JS, et al. Leadless pacemaker versus transvenous single-chamber pacemaker therapy: a propensity score-matched analysis. Heart Rhythm. 2018. 10.1016/j.hrthm.2018.04.027. 10.1016/j.hrthm.2018.04.027 29709576

[57] Chinitz L, Ritter P, Khelae SK, Iacopino S, Garweg C, Grazia-Bongiorni M, et al. Accelerometer-based atrioventricular synchronous pacing with a ventricular leadless pacemaker: results from the Micra atrioventricular feasibility studies. Heart Rhythm. 2018. 10.1016/j.hrthm.2018.05.004. 10.1016/j.hrthm.2018.05.004 29758405

[58] Karim S, Abdelmessih M, Marieb M, Reiner E, Grubman E (2016). Extraction of a Micra Transcatheter Pacing System: first-in-human experience. HeartRhythm Case Rep.

[59] Omdahl P, Eggen MD, Bonner MD, Iaizzo PA, Wika K (2016). Right ventricular anatomy can accommodate multiple Micra transcatheter pacemakers. Pacing Clin Electrophysiol.

[60] Hartig F, Köhler A, Stühlinger M (2018). Carotid sinus syndrome: a case report of an unusual presentation of cardiac arrest while diving. European Heart Journal - Case Reports.

[61] Kypta A, Blessberger H, Lichtenauer M, Kammler J, Lambert T, Kellermair J (2016). Subcutaneous double "purse string suture"-a safe method for femoral vein access site closure after leadless pacemaker implantation. Pacing Clin Electrophysiol.

[62] Grubman E, Ritter P, Ellis CR, Giocondo M, Augostini R, Neuzil P (2017). To retrieve, or not to retrieve: system revisions with the Micra transcatheter pacemaker. Heart Rhythm.

[63] Afzal MR, Daoud EG, Cunnane R, Mulpuru SK, Koay A, Hussain A, Omar R, Wei KK, Amin A, Kidwell G, Patel N, Love C, Lloyd M, Sterliński M, Goldbarg S, Leal MA, Gabriels J, Patel A, Jadonath R, Grubman E, Crossley G, Pepper C, Lakkireddy D, Okabe T, Hummel JD, Augostini RS (2018). Techniques for successful early retrieval of the Micra transcatheter Pacing System: a worldwide experience. Heart Rhythm.

[64] Kusumoto FM, Schoenfeld MH, Wilkoff BL, Berul CI, Birgersdotter-Green UM, Carrillo R, Cha YM, Clancy J, Deharo JC, Ellenbogen KA, Exner D, Hussein AA, Kennergren C, Krahn A, Lee R, Love CJ, Madden RA, Mazzetti HA, Moore JC, Parsonnet J, Patton KK, Rozner MA, Selzman KA, Shoda M, Srivathsan K, Strathmore NF, Swerdlow CD, Tompkins C, Wazni O (2017). 2017 HRS expert consensus statement on cardiovascular implantable electronic device lead management and extraction. Heart Rhythm.

